# Physiochemical Characterization and Release Rate Studies of SolidDispersions of Ketoconazole with Pluronic F127 and PVP K-30

**Published:** 2011

**Authors:** Pankaj Kumar, Chander Mohan, Mara KanamSrinivasan Uma Shankar, Monica Gulati

**Affiliations:** a *Department of Pharmaceutical Sciences, Lovely Professional University, Chaharu, Phagwara, Punjab-144402, India.*; b *Rayat College of Pharmacy, Hoshiarpur, Punjab, India.*

**Keywords:** Solid dispersion, Ketoconazole, PVP K-30, Pluronic F127, Solvent evaporation, Melt-fusion

## Abstract

In the present study solid dispersions of the antifungal drug Ketoconazole were prepared with Pluronic F-127 and PVP K-30 with an intention to improve its dissolution properties. Investigations of the properties of the dispersions were performed using release studies, Differential scanning calorimetery (DSC), X-ray powder diffraction (XRD) and Fourier transform infrared (FTIR). The results obtained showed that the rate of dissolution of Ketoconazole was considerably improved when formulated in solid dispersions with PVP K-30 and Pluronic F-127 as compared with pure drug and physical mixtures. The results from DSC and XRD studies showed the transition of crystalline nature of drug to amorphous form, while FTIR studies demonstrated the absence of drug-carriers interaction.

## Introduction

Ketoconazole, is a member of imidazole containing compound that is used as a broad spectrum antifungal agent for the treatment or prevention of fungal infections especially against thrush, gastrointestinal (GI) infections, and infections of the skin, nails, and scalp. Ketoconazole has oral tablet, cream and dandruff shampoo formulations.

Ketoconazole is the member of imidazole class that is currently used in the treatment of systemic infections (1). Ketoconazole is classified in the Biopharmaceutics Classification Scheme (BCS) as a class II drug, since it has a high permeability and its solubility in aqueous media is not sufficient for the whole dose to be dissolved in the GI fluids under normal conditions (2) Although many solubilization techniques have been described that either changes the nature of solvent environment (co-solvents systems, emulsions, micellization) or the chemical identity of the desired solute (salt formation, prodrugs); however, in comparison drugs into hydrophilic carriers is an alternate option for improving the drug bioavailability (3-5) Such dosage forms are referred to as solid dispersions; they are defined as the dispersion of one or more active ingredients in an inert hydrophilic carrier or matrix at solid state prepared by the fusion, solvent or solvent–fusion method (6, 7) This system provides the possibility of reducing the particle size of drugs to nearly a molecular level, to transform the drug from the crystalline to the (partial) amorphous state and/or to locally increase the saturation solubility. They can form the basis of products applied for various routes of administration and for various dosage forms, including the most popular dosage form; the tablet.

Vasconcelos*et al*. (8) classified solid dispersions into three categories *i.e*. first generation (crystalline carriers), second generation (amorphous carriers) and third generation (surfactants, mixture of surfactants and polymer with surfactants). In second generation solid dispersions, the drug is in its supersaturated state because of forced solubilization in the carrier (9-12) while in first generation drug is entrapped in crystalline carriers so drug is also not completely amorphous in nature. These systems are able to reduce the drug particle size to nearly a molecular level, to solubilize or co-dissolve the drug by the water-soluble carrier, to provide better wettability and dispersibility of the drug by the carrier material, and to produce amorphous forms of the drug and carriers (13, 14). Due to the limited solubilizing capability of carriers, various pharmaceutical excipients, such as solubilizers, surfactants, oils and fatty acids, or in the form of mixtures, can be added into the solid dispersions to further improve the drug solubility and dissolution rate (15-19) as in third generation SD. The use of surfactants such as inulin (20), inutec SP1 (21), compritol 888 ATO (22), gelucire 44/14 (14) and poloxamer 407 (23), as carriers, was shown to be effective in originating high polymorphic purity and enhanced *in-vivo *bioavailability. The inclusion of surfactants in the formulation containing a polymeric carrier may help prevent precipitation and/or protect a fine crystalline precipitate from agglomeration into much larger hydrophobic particles (24). These solid dispersions are intended to achieve the highest degree of bioavailability for poorly soluble drugs and to stabilize the solid dispersion (SD), avoiding drug recrystallization (8). The aim of present study was to prepare and characterize different solid dispersions of ketoconazole with PVP K-30 and Pluronic F127 so as to improve its dissolution properties.

## Experimental

Ketoconazole was obtained as a gift sample from Yash Pharmaceuticals, Roorkee, India. Pluronic F127 was provided as gift sample by Signet Chemicals, India and its melting point varies from 52 to 57°C. PVP K-30 was procured from Central Drug House, New Delhi, India and gelatin capsule shells (000 sizes) were purchased from Saini Medical Hall, Punjab, India. All the materials used in the study were of analytical grades.

In order to evaluate the effect of carriers on Ketoconazole, phase solubility studies and dissolution studies were performed. Physical analysis based on DSC, FTIR, and XRD was performed to evaluate the structure of the dispersions and to detect the possible drug-carrier interactions.

*Phase solubility studies*

Phase solubility studies were performed (method proposed by Higuchi and Connors) (25) by adding an excess of Ketoconazole to 50 mL volumetric flasks containing 10 mL triple distilled water, phosphate buffer pH 6.8 and aqueous carrier solutions (1%, 2%, 4%, 6% and 8% w/v concentration range) of Pluronic F127 and PVP K-30. Volumetric flasks were shaken on water bath incubator shaker (Navyug Q-5247, India) at 30 ± 0.5°C for 48 h. At equilibrium after 48 h, the suspension was filtered through whatman filter paper (0.45 μ pore size) and aliquots were withdrawn to determine drug content spectrophotometrically at λ_max_244.8 nm (Systronics UV 2203 Spectro-photometer, Ahmedabad, India).

*Preparation of physical mixtures*

Physical mixtures (PMs) of ketoconazole were prepared by mixing drug with carrier in a mortar (26) until homogenous mixture was obtained. The resulting mixture were sieved through a 355 μm mesh and then stored in a desiccator at room temperature until use. As Pluronic F127 is a crystalline carrier, it was first crushed and then passed through 355 μm mesh before mixing with drug.

*Preparation of solid dispersions formulations*

*Melt-fusion method*

As Pluronic F127 has a melting point range of 52-57°C (27), solid dispersions were prepared by melt-fusion method with different concentrations of Ketoconazole (1:1, 1:2, 1:4, 1:6, and 1:8) and through melting physical mixture of drug and Pluronic F127 on a water bath at 90°C with continuous stirring and by rapidly cooling the resulting homogeneous preparations rapidly cooled over ice bath (28, 29) Subsequently, the dispersions were pulverized, passed through a 355 μm sieve, and then stored in a desiccator at room temperature until use.

*Solvent evaporation method*

As PVP K-30 is soluble or sparingly soluble in a wide range of commonly used solvents like methanol, ethanol, chloroform, dichloromethane *etc*. (27), therefore, solvent evaporation method is suitable technique for preparing solid dispersions of drug with PVP K-30. Chloroform was used as a common solvent for preparing solid dispersions. Five different preparations of Ketoconazole with PVP K-30 were prepared in the drug to carrier ratios of 1:1, 1:2, 1:4, 1:6, and 1:8 respectively. Ketoconazole and carrier were dissolved in chloroform subsequently placed in a vacuum oven (Navyug Q-5247, India) at 15 lb pressure, 40°C temperature for 72 h until formulations appeared in a glassy transparent stage without any moisture or stickiness and were stored in dessicator for further 48 h. Subsequently the dispersions were pulverized, passed through a 355 μm mesh and stored in dessicator at room temperature until use.

*Dissolution studies*

Dissolution rate studies were performed in 900 mL phosphate buffer (pH 6.8) at 37 ± 0.1°C; using single stage USP XXII apparatus (paddle method, 100 rpm). Pure Ketoconazole drug, solid dispersions as well as physical mixtures, each containing equivalent to 100 mg drug were filled in empty hard gelatin capsule shells and subjected to dissolution studies. At fixed time intervals (10, 20, 30, 45, 60, 90, 120 min) aliquots of 5 mL samples were withdrawn and simultaneously replenished with fresh 5 mL of phosphate buffer solution maintained at same temperature to maintain sink conditions. Samples, so withdrawn were immediately filtered through whatman filter paper (pore size 0.45μ) and assayed spectrophotometrically for drug content at 244.8 nm.

*Content uniformity*

Precisely weighed amounts of solid dispersions equivalent to 20 mg of Ketoconazole was dissolved in 50 mL methanol. The drug content was determined spectophotometrically with appropriate dilutions from methanol at 244.8 nm.

*X-Ray diffraction study*

Powder X-ray diffraction (XRD) can be used to qualitatively detect material with long range order. Sharper diffraction peaks indicate more crystalline material. The crystallinity of the pure drug, carriers and solid dispersions were measured using a XPERT-PRO apparatus exposed to CuKα (λ = 1.54060 Å) radiation (45 kV X 40 mA) from a PW3064 X-ray generator. The degree of diffractions was measured at 10°/min every 0.017° between 5º and 45º (2θ). Samples were prepared into aluminum frames. For the preparation, the front of the frames was mounted on a smooth teflon plate. The samples were filled into the window and were compressed with a slide. This procedure avoideda preferential orientation of the particles. Silicon was used as an internal standard.

*Differential scanning calorimetry study*

Differential scanning calorimetry (DSC) measurements were carried out of the Ketoconazole, carriers and solid dispersion formulations which showed better release rate from dissolution studies using a DSC Q10 V9.9 (Build 303, Germany). Samples of approximately 3-4 mg were sealed in pierced aluminum pans of 40 μL and were measured at a scanning speed of 20°C /min over a temperature range from 40-200°C. The samples were cycled twice to remove the effects of moisture and thermal history. The solid-state characteristics were surveyed in the heating cycle by observing the melting enthalpy and the onset melting temperature of the drug. An empty pan served as reference.

*Fourier transform infrared spectroscopy*

Fourier transform infrared spectroscopy (FTIR) spectra were obtained on a Shimadzu 8400S IR solution 1.30 system, (Shimadzu Corporation) using potassium bromide (KBr) disc method. To avoid the effect of moisture, all samples were dried overnight in a dessicator. The FTIR spectra, in transmittance mode, were obtained in the spectral region of 4000-400 cm^-1^ using a resolution of 4 cm^-1^ and 20 scans. A polystyrene filter was used to check the spectrophotometer calibration and KBr pellet was used as reference.

## Results and Discussion

*Phase solubility studies*

**Figure 1:**
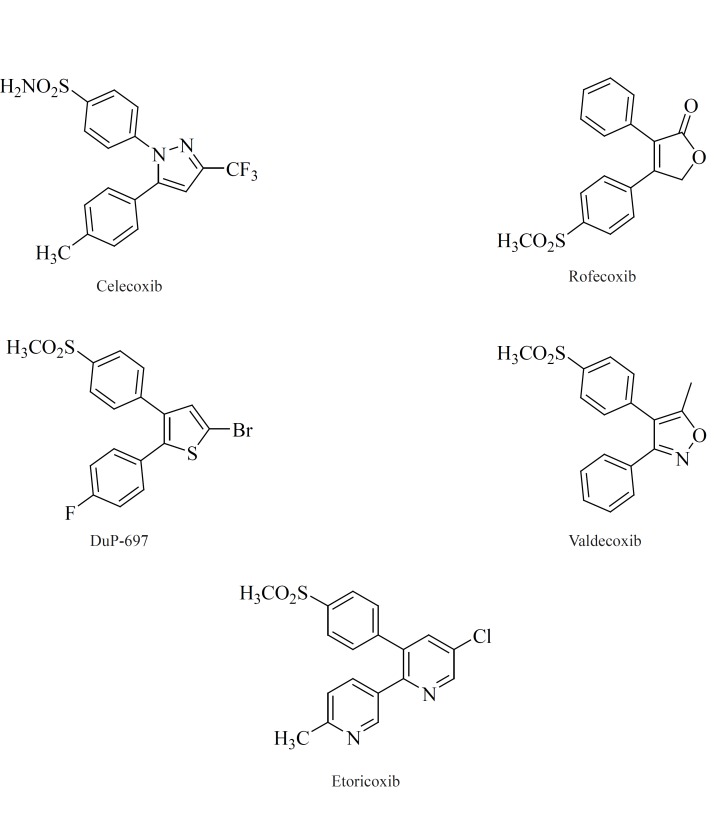
Chemical structure of Ketoconazole

The solubility of Ketoconazole in water at 30°C was found to be 8.383 μg/mL; therefore, Ketoconazole is considered as a practically insoluble drug. The phase solubility diagram obtained for ketoconazole solutions of Pluronic F127 or PVP K-30 at 30°C is shown in Figure 1.

 The increase in solubility was linear with respect to weight fraction of the carrier and increase in solubility at 30°C was about 54-fold and 65-fold with Pluronic F127 and PVP K-30 respectively compared to pure Ketoconazole. The increase in solubility in the presence of carrier can probably be explained by increased wettability of Ketoconazole and micellarsolubilization. Indeed, Pluronic F127 causes a decrease of interfacial tension between drug and the dissolving solution.

Table 1 presents the thermodynamic parameters obtained with aqueous solubility of Ketoconazole in the presence of Pluronic F127 or PVP K-30.

**Table 1:** Thermodynamic parameters of the solubility process of Ketoconazole in water-carriers system

**% Carrier**	**ΔG**_tr_ º (KJ/mol) ± SD*	
	**Pluronic F127**	**PVP K-30**
1	-0.395	-0.401
2	-0.474	-0.404
4	-0.616	-0.573
6	-0.730	-0.763
8	-0.821	-0.857

* Data are expressed as mean ± SD (n = 3)

 The indication of process transfer of Ketoconazole to different concentrations of aqueous solution of PVP K-30 and Pluronic F127 may be obtained from the values of Gibbs free energy change (30, 31). The Gibbs free energy of transfer (ΔG_tr_º) of Ketoconazole from water to aqueous solutions of carriers was calculated using the following equation:

ΔGtrº = -2.303 RT log (Sc/So) 

Where ΔG_tr_º is Gibbs free energy of transfer, R (8.314 J/°C mol) is gas rate constant, T is temperature (30°C) at which phase solubility studies were conducted and S_c_/S_o _is the ratio of molar solubility of Ketoconazole in aqueous solution of carriers to that of water. The acquired values of ΔG_tr_º indicate whether the drug solubilization in the aqueous solution is favorable or not i.e. negative ΔG_tr_º values indicate favorable conditions and when the values decreases more; means more favorable conditions. The data’s provided the information regarding increased solubility of Ketoconazole in presence of Pluronic F127 and PVP K-30; ΔG_tr_º values were all negative indicating the spontaneous nature of drug solubilization and it decreased with increase in carriers concentration demonstrating that the reaction became more favorable as concentration of carriers increased.

*Dissolution studies*

**Figure 2:**
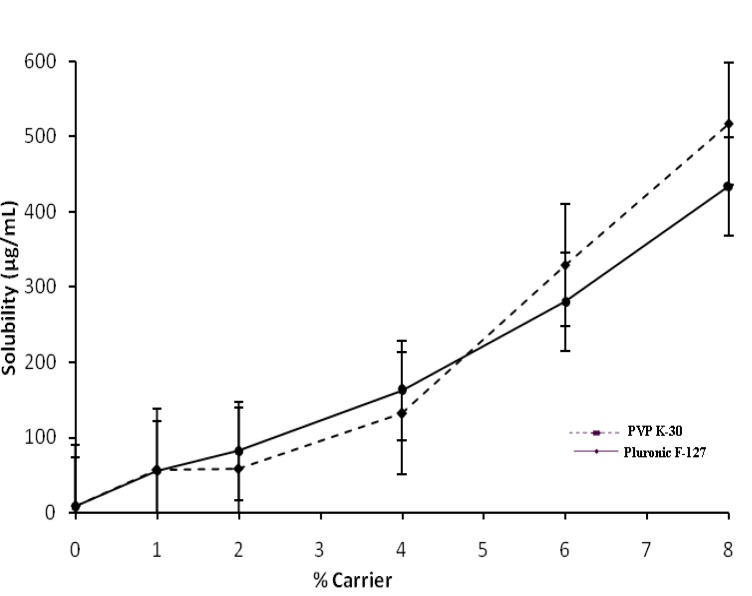
Phase solubility diagram of Ketoconazole in aqueous solutions of PVP K-30 and Pluronic F-127 at 30^°^C; Error bars indicate the standard deviation, n = 3

**Figure 3:**
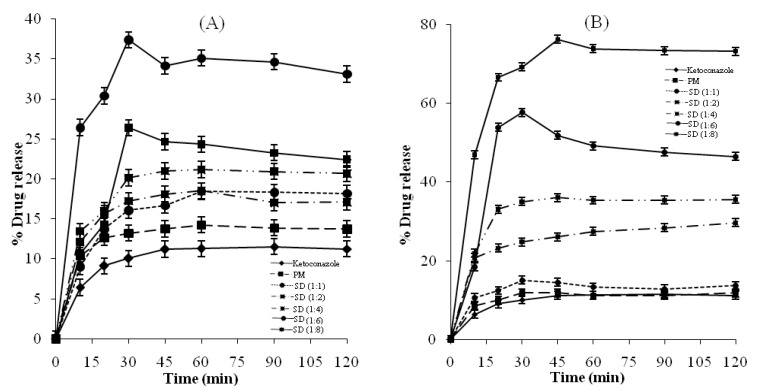
.(A) Dissolution profiles of Ketoconazole, physical mixture (PM) and solid dispersions (SDs) of Ketoconazole with Pluronic F-127, (B) Dissolution profiles of Ketoconazole, physical mixture (PM) and solid dispersions (SDs) of Ketoconazole with PVP K-30

Figures 2 and 3 show the amount of Ketoconazole dissolved as a function of time from physical mixtures and solid dispersions of Ketoconazole with Pluronic F127 and PVP K-30 respectively. It is evident that the rate of dissolution of pure Ketoconazole is very low, less than 12% of the drug being dissolved within 3 h. Dispersion of Ketoconazole in the hydrophilic carriers considerably enhanced dissolution (36-68%) compared to the physical mixtures (12-14%). The dissolution of the physical mixtures was higher compared to pure Ketoconazole. Possible mechanisms of increased dissolution rates of solid dispersions have been proposed by Craig, (32) and include: reduction of drug crystallite size, a solubilisation effect of the carrier, absence of aggregation of drug crystallites, improved wettability and dispersibility of a drug from the dispersion, dissolution of the drug in the hydrophilic carrier, conversion of the drug to the amorphous state and finally the combination of the above mentioned methods. Dry mixing of Ketoconazole with PVP or Pluronic F127 brings drug in close contact with the hydrophilic carrier. The increased dissolution rate in this study can thus be contributed by several factors such as a solubilisation effect of the carrier, conversion of drug to amorphous state, improved wettability and dispersibility of the drug.

*Drug content in polymeric solid dispersions*

The content of Ketoconazole in solid dispersions prepared with various concentrations of Pluronic F127 and PVP K-30 have been shown in Table 2. Percentage drug contents ranged from 99.8 to 102.6% in solid dispersions prepared from Pluronic while in case of solid dispersions of PVP K-30 it ranged from 99.4 to 102.9%.

**Table 2:** Percentage Drug contents in solid dispersions

**SD ratios**	% Drug content ± SD*	
	**Pluronic F127**	**PVP K-30**
1:1	99.8 ± 0.2	99.4 ± 0.5
1:2	102.3 ± 0.3	99.9 ± 0.3
1:4	101.2 ± 0.1	102.7 ± 0.2
1:6	101.0 ± 0.4	101.6 ± 0.3
1:8	103.0 ± 0.1	100.2 ± 0.5

* Data are expressed as mean ± SD (n = 3).

*Powder X-Ray diffraction*

**Figure 4:**
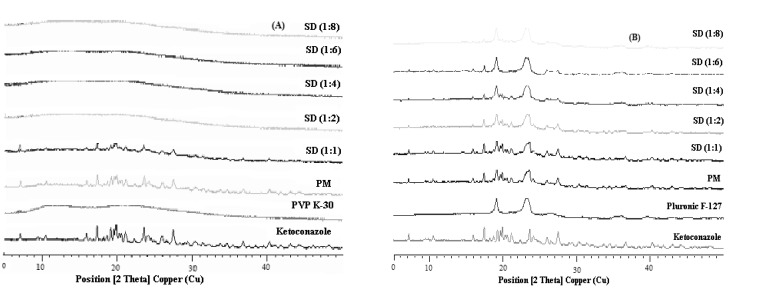
**.**(A) X-ray diffraction patterns of solid dispersions (SDs) and physical mixture (PM) of Ketoconazole with PVP K-30, (B) X-ray diffraction patterns of solid dispersions (SDs) and physical mixture (PM) of Ketoconazole with Pluronic F-127

Figure 4 show the X-ray diffractograms for Ketoconazole, PVP K-30, Pluronic F127, PMs and solid dispersions investigated. The diffraction spectrum of pure Ketoconazole showed that the drug was crystalline in nature as demonstrated by numerous distinct peaks notably at 2θ angles 19.9°, 17.4°, 23.6° and 27.5°. The characteristic peaks for Ketoconazole and their relative intensities are presented in Table 3. Pluronic F127 also showed two characteristic peaks with the highest intensity at 2θ angles 19.13° and 23.32° indicating that Pluronic is a crystalline carrier. Upon increasing the concentration of the carriers, the peaks from Ketoconazole became less intense. The extent of crystallinity of the phases will influence the dissolution of the dosage forms. An amorphous or metastable form will dissolve at the fastest rate because of its higher internal energy and greater molecular motion which enhances the thermodynamic properties relative to crystalline materials (33).

**Table 3:** Intensities at characteristic diffraction angles 2θ and d-values (Å) for Ketoconazole

**2θ**	**d-spacing [Å]**	**Rel. Int. [%]**	**2θ**	**d-spacing [Å]**	**Rel. Int. [%]**
7.190	12.294	33.21	24.149	3.685	33.95
9.408	9.4002	10.73	25.134	3.543	10.22
10.508	8.4185	25.37	25.979	3.429	34.07
11.973	7.3914	11.36	26.509	3.362	13.81
14.727	6.0150	10.61	27.476	3.246	66.27
15.933	5.5624	39.10	29.366	3.041	11.95
17.416	5.0919	91.00	30.276	2.952	14.16
18.695	4.7464	29.36	34.525	2.597	10.08
19.905	4.4604	100.00	36.645	2.452	17.08
21.159	4.1988	46.02	36.645	2.452	17.08
22.286	3.9889	15.29	40.256	2.240	16.48
23.608	3.7686	80.11			

Based on our findings, it is clear that Ketoconazole was converted to the amorphous form in solid dispersions of PVP K-30 while drug is not completely amorphous in solid dispersions of Pluronic F127. All the principal peaks from Pluronic F127 and Ketoconazole were present in their respective physical mixtures and solid dispersions, although with lower intensity, but no new peaks could be observed, suggesting the absence of interaction between the drug and the carrier. The shift of the intensity of Ketoconazolepeaks observed in solid dispersions with Pluronic F127 as compared to their respective physical mixtures can be explained as a result of change of crystal orientation. Likewise, the peak at 2θ from Ketoconazole progressively decreased in intensity as the concentration of Pluronic F127 was increased in both physical mixtures and solid dispersions. The intensity of the peaks from Ketoconazole was highly reduced in PVP K-30 preparations at 2θ angles and these were completely absent at higher ratios of PVP K-30 solid dispersions. From these observations it can be deduced that the crystalline nature of the drug was still maintained but the relative reduction of diffraction intensity of Ketoconazole peaks in Pluronic F127 preparations at these angles suggest that the quality of the crystals became worse. The positions of Pluronic F127 patterns in the physical mixtures and solid dispersions were the same and super-imposable, which again ruled out the possibility of chemical interaction and compound formation between these two components. In the case of PVP K-30 preparations, d-values were absent which is due to the possible formation of glass solutions. Glass solution formation involves the complete disappearance of drug peaks in the preparation which was observed at higher ratios of solid dispersions from PVP K-30.

*Differential scanning calorimetry (DSC)*

**Figure 5:**
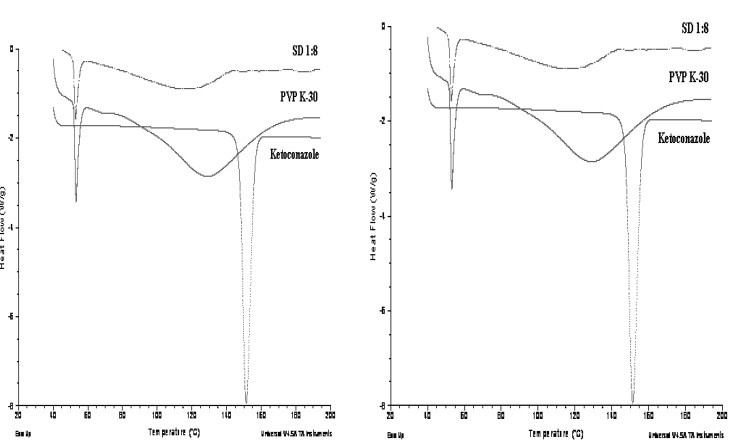
**.**(a) DSC thermograms of pure drug, Pluronic F-127 and solid dispersion (1 : 6) of Ketoconazole in Pluronic F-127 at a scanning rate of 20°C; (b) DSC thermograms of pure drug, PVP K30 and solid dispersion (1:8) of Ketoconazole in PVP K-30 at a scanning rate of 20°C

DSC curves obtained for pure Ketoconazole,Pluronic F127 and the solid dispersions (1:6) prepared with Pluronic F127 has shown in Figure 5. 

Pure powdered Ketoconazole and Pluronic F127 showed a sharp melting endothermic peak (T_m_) at 151.35°C and 56.30°C respectively while PVP K-30 showed broad glass transition (T_g_) peak at 128.69°C. Solid dispersion prepared from Pluronic F127 showed reduced and a slightly broad peak of drug at 123.06°C and peak of crystalline carrier was also shifted slightly but showed sharp peak at 55.33°C. Solid dispersion showed no crystalline peak of ketoconazole and a slightly broad and shifted peak of PVP K-30 at 114.96°C. This implies from DSC data that the drug is present in crystalline form in Pluronic F127 solid dispersion but the crystallinity has been reduced as compared to pure drug while drug is present in completely amorphous form in PVP K-30 solid dispersion.

*FTIR analysis*

**Figure 6:**
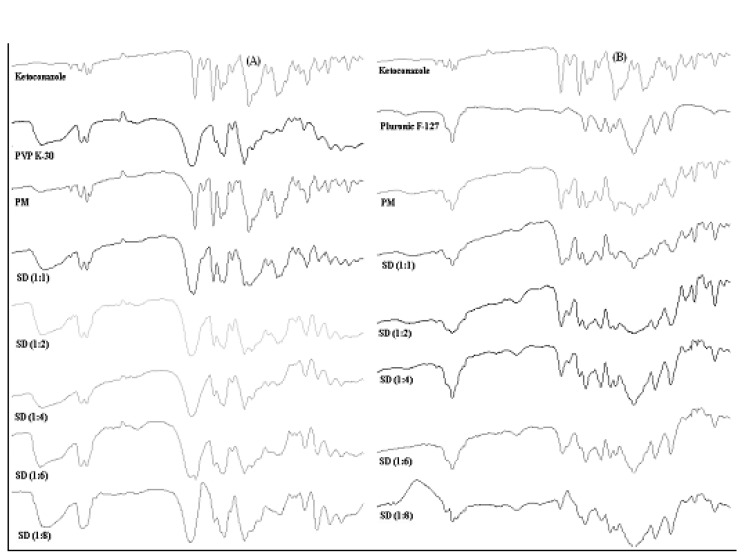
(A) FTIR-spectroscopy of binary solid dispersions and physical mixtures of Ketoconazole with PVP K-30; (B) FTIR-spectroscopy of binary solid dispersions and physical mixtures of Ketoconazole with Pluronic F-127

In order to further study the possibility of an interaction of Ketoconazole with PVP K-30 and Pluronic F127 in the solid state, more information was gathered using FTIR spectroscopy (Figure 6). 

Pure ketoconazole displayed characteristic peaks of C=O stretching vibration of carbonyl group, C-O stretching of aliphatic ether group and C-O stretching of cyclic ether at 1647.26 cm^-1^, 1031.95 cm^-1^ and 1244.13 cm^-1^ respectively. If the drug and the polymer would interact, then the functional groups in the FTIR spectra would show band shifts and broadening compared to the spectra of the pure drug and polymer (34). The FTIR spectra obtained from the various solid dispersions showed peaks which were a summation of the characteristic peaks obtained with the pure drug and pure carriers and spectra’s can be simply regarded as the superposition of those of Ketoconazole and carriers. This showed that there was no chemical interaction of the drug with carriers even in the amorphous state when the granules were prepared by the solid dispersion method. An increase in the polymer content also did not initiate any drug polymer interactions. FTIR spectra indicated reduction in sharpness of peaks in solid dispersions as compared to pure drug as well as physical mixtures. It implied from FTIR that due to reduction in sharpness crystallinty of Ketoconazole was reduced in solid dispersions (35) as already confirmed from X-ray diffraction and DSC.

## Conclusions

From the results of this study we were able to show that the formulation of PVP K-30 and Pluronic F127 markedly improves its dissolution properties. Solid dispersions demonstrated a higher dissolution rate than physical mixtures and pure drug. X-ray powder diffraction and DSC indicated that the drug was completely amorphous when dispersed in PVP K-30 from SD 1 : 2 and thereafter ratios while in solid dispersions of Pluronic F127 only reduction in crystalline nature of drug was observed but drug was not completely amorphous. The increased dissolution rate in systems containing Pluronic F127 and PVP K-30 was probably due to transition of crystalline nature of drug in solid dispersions, reduction in particle size, increased wettability and dispersibility of ketoconazole, since no interactions in the solid state could be demonstrated. Solubility studies showed a solubilizing effect of both carriers on ketoconazole. The negative values of the Gibbs free energy indicated the formation of solid dispersion is favorable condition. On comparison the two formulations second generation amorphous carrier (PVP K-30) has better dissolution rate and solubilizing action on ketoconazole than third generation (Pluronic F127) crystalline carrier with surface active property. Thus, the formulation of solid dispersions of a drug with hydrophilic carriers is a potential approach used to improve the solubility and dissolution rate of practically water-insoluble or less soluble drugs since almost last five decades.
